# Advanced analysis and visualization of gene copy number and expression data

**DOI:** 10.1186/1471-2105-10-S1-S70

**Published:** 2009-01-30

**Authors:** Reija Autio, Matti Saarela, Anna-Kaarina Järvinen, Sampsa Hautaniemi, Jaakko Astola

**Affiliations:** 1 Department of Signal Processing, Tampere University of Technology, 33101, Tampere, Finland; 2 Biomedicum Biochip Center and Institute of Biomedicine, 00014 University of Helsinki, Helsinki, Finland; 3 Computational Systems Biology Laboratory, Institute of Biomedicine and Genome-Scale Biology Research Program, 00014 University of Helsinki, Helsinki, Finland

## Abstract

**Background:**

Gene copy number and gene expression values play important roles in cancer initiation and progression. Both can be measured with high-throughput microarrays and some methodologies to integrate and analyze these data exist. However, varying gene sets within different gene expression and copy number microarrays present significant challenges.

**Results:**

We report an advanced version of earlier published CGH-Plotter that rapidly can identify amplified and deleted areas using gene copy number data. With CGH-Plotter v2, the copy number values can be filtered based on the genomic location in basepair units. After filtering, the values for the missing genes can be interpolated. Moreover, the effect of non-informative areas in the genome can be systematically removed by smoothing and interpolating. Further, we developed a tool (ECN) to illustrate the CGH-data values annotated based on the gene expression. The ECN-tool is a MATLAB toolbox enabling straightforward illustration of copy numbers annotated based on the gene expression levels.

**Conclusion:**

CGH-Plotter v2 provides two methods for analyzing copy number data; dynamic programming and genomic location based smoothing. With ECN-tool the data analyzed with CGH-Plotter v2 can easily be illustrated along the chromosomes individually or along the whole genome. ECN-tool plots the copy number data annotated based on the gene expression data, and it is easy to find the genes that are both over-expressed and amplified or under-expressed and deleted in the samples. From the resulting figures it is straightforward to select interesting genes.

## Background

Gene copy numbers as well as gene expression values play an important role in biomedical research. Especially in cancer research both are often on focus. In particular, it is very important to find genes that are both over-expressed and amplified, or under-expressed and deleted because these genes may be over- or under-expressed in cancers due to large chromosomal instabilities that are common in various cancers.

Copy number changes are common in cancer, and they are known to involve genes that play a crucial role in the development and progression of this malignant disease [1]. These amplifications and deletions often happen for a larger part of the genome spanning over several genes at the same time. With array-CGH (Comparative Genomic Hybridization) the copy number values of the genes can be measured [2]. In a CGH microarray experiment, strands of DNA are hybridized to the slide and copy numbers of thousands of genes can be measured simultaneously [3].

The alterations in the gene copy numbers have been associated with aberrant gene expression [4]. In many cases, both the gene copy numbers, and the gene expression values of the sample are analyzed with DNA microarrays [5,6]. In general, the microarrays used to measure either copy number or gene expression data are different, which hinders integration of the values.

We have earlier developed CGH-Plotter – the first toolbox for systematic array-CGH data analysis and illustration, with which the amplified and deleted regions within a genome can be identified [7]. In addition to CGH-Plotter, there are now several methods for analyzing copy number data, especially for finding the copy number alterations, amplifications and deletions. For example, M-CGH uses first nearest neighbour method for smoothing the data, then fuzzy k-nearest neighbour to classify the data to three classes, gain, normal and loss [8]. Often, the aberrations are sought with smoothing algorithms [9,10]. Additionally, unsupervised hidden Markov models approach [11] and circular binary segmentation [12] have been used for identifying the alterations in the copy numbers of genes. Thus, a widely-used tool CGHPRO provides both of them with several additional smoothing possibilities for analyzing the CGH data [13]. There are not many options to analyze and illustrate the copy number and expression data together. However, one option is ISACGH, a web based interface, with which the gene expression and copy number values can be studied together [14].

## Results

**Figure 1:**
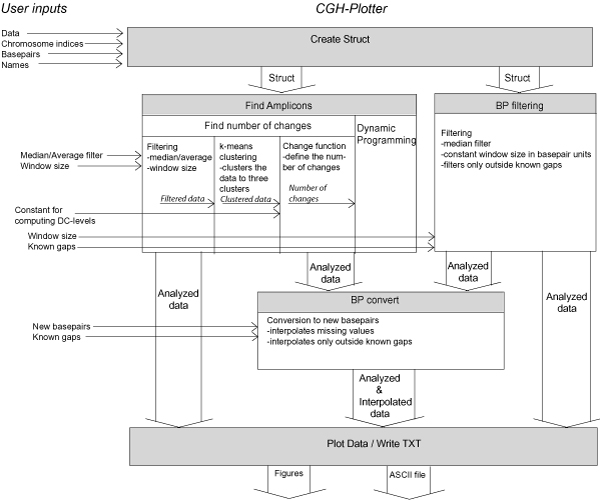
**Overall view of CGH-Plotter v2**. CGH data, chromosome indices, basepairs and names of the samples are input into CGH-Plotter. Further, the type of the filter and the size of the window, which are used in filtering phase, are inputs if constant levels are calculated with dynamic programming algorithm. CGH-Plotter clusters the filtered data into three clusters with k-means clustering algorithm. Clustered data are delivered to the function, which computes the maximum number of the change points. The number of changes is needed when dynamic programming algorithm computes the gains and losses [7]. Other option is to smooth the data with basepair location based filter. Known gaps and the window size for filtering needs to be input in basepair units. Further, if interpolation wanted to take place, new basepair locations for genes with need for copy number value has to be given to CGH-Plotter. The results of the analyses can be plotted and saved into ASCII-file.

CGH-Plotter is a MATLAB toolbox for detecting amplified and deleted areas within the CGH data [7]. Here we present modifications to CGH-Plotter, which improve its performance in CGH data analysis, and provide new possibilities for analyzing gene copy number data (Figure 1).

Original version of CGH-Plotter analyzes the copy number values with three step function that included filtering, k-means clustering and dynamic programming [7]. In addition, CGH-Plotter offers several options for plotting the data. However, in several collaborations we identified that the genomic location should be taken into account more also in the analysis step in addition to visualization. Therefore, we added a possibility to smooth the data based on the cumulative basepair location of the genes. Further, often the copy numbers of the genes are compared to the other measures of the genes, such as gene expression values.

Since often the microarrays used in gene expression measurements are different from the microarrays used in CGH measurements, the direct comparison between the data values is challenging. Therefore, we added a possibility to obtain the copy numbers of the genes not present in the array in use. The assumption behind this function is that the number of the genes in the array is dense enough to give the copy number levels of regions in the genome.

### Genomic location based gene value smoothing

Cumulative basepairs define the locations of the genes. In CGH-Plotter v2, the copy number data can be smoothed based on basepair indicated genomic location. CGH-Plotter utilizes moving window median or mean filter with a constant window size in basepair units. The user may select the size of the window in basepair units for filtering the data.

In the real CGH data, there are always locations, such as centromeres and telomeres, where adjacent genes should not interact with each other during the filtering process. Therefore, we have added a possibility to give CGH-Plotter the regions that should be treated separately during the filtering process. With these regions we are able to filter the data in a more reliable fashion.

The moving window for smoothing is defined by a genomic distance around each gene instead of the number of genes. Moreover, known gaps in the genome, such as telomeres, centromeres, and heterochromatin regions, can be taken into account, and the genes on different sides of these areas will not be included into the same window in filtering. Thus, a file of all the areas including genetic information needs to be input to the CGH-Plotter and the filtering will be done based on these areas. The filter moves along each chromosome and selects the window around the genes based on the given window size and gaps in the genome.

### Genomic location based gene value interpolation

There are also cases where interesting clones of the genome are not observed, or the clones are not included in the microarray at all. Therefore we have added a possibility to add clones to the filtered data. Since the amplifications and deletions span large regions, we are able to estimate the values of the missing genes based on the filtered copy number ratios of the genes next to the missing one. The values for these missing clones are determined with linear interpolation.

### Illustration of CGH and gene expression values

The results of the CGH-Plotter can be further illustrated and annotated based on the gene expression values with the Expression annotation of Copy Number (ECN)-tool. The ECN-tool is a MATLAB toolbox enabling straightforward illustration of copy numbers annotated based on the gene expression levels.

**Figure 2:**
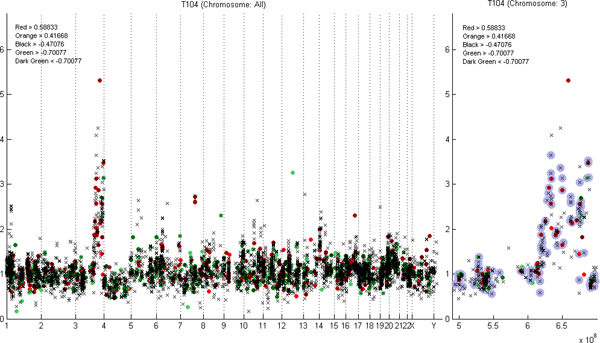
**Example plots of ECN-tool**. On left, the gene copy number levels (y-axis) of all chromosomes of the sample T104 are plotted against genomic location in basepair units (x-axis) annotated based on the gene expression levels. Over-expressed genes are annotated in red and under-expressed genes with green. The boundaries of colors indicating the expression levels are explained in the left top corner of the figure. On right, the values of the chromosome 3 of the same sample T104 are plotted against the cumulative basepairs. Interpolated values are indicated with grey circles around the spots.

Together with expression data, CGH data can be plotted along the chromosomes individually or along whole genome (Figure 2). Thus, it is easy to find the genes that are both over-expressed and amplified in the sample. From this user interface plot, it is straightforward to save the copy number values and gene expression values of the genes to a text file. All the values of the sample can be imported to a text file (Table 1). Another possibility is to select manually the interesting genes to be written into the text file. In the resulting plot, interpolated copy numbers are annotated separately, and thus it is easy to see whether the value is real or interpolated based on the values of the neighbours.

**Table 1:** Resulted txt-file

Gene name	Basepair	EXP	CGH	Filtered CGH	Interpolated
ENSG00000187583	891740	NaN	NaN	0,0452	1
ENSG00000187642	900447	0,0306	NaN	0,0452	1
ENSG00000188290	924207	-0,1220	NaN	0,0452	1
ENSG00000187608	938666	-0,2577	0,9464	0,0452	0
ENSG00000188157	945366	-0,4694	0,8683	0,0452	0
ENSG00000131591	1007061	-0,7750	NaN	0,0362	1
ENSG00000186891	1128751	0,3375	NaN	0,0184	1
ENSG00000186827	1136569	-0,1087	NaN	0,0172	1
ENSG00000078808	1142151	-0,3429	NaN	0,0164	1
ENSG00000176022	1157508	-0,5600	NaN	0,0142	1
ENSG00000160087	1179157	0,1557	1,5931	0,0110	0
ENSG00000162572	1205831	-0,3048	1,4057	-0,0221	0

First twelve rows of the text file into which the results plotted in Figure 2 are written. All the data values can be written into the text file. In this example, user has input the Ensemble names for the genes [18]. In addition cumulative basepair location, logarithmic expression value, CGH ratio, and logarithmic filtered CGH value is given. Many of the values in CGH column are NaNs, which means that the values are not detected in the CGH microarray. The last column reveals whether the filtered value is interpolated or not.

### Case study

In our test case, there were CGH and gene expression values from together 38 samples of which 20 samples are head and neck cancer data [15] and 18 samples oral tongue cancer data [16]. We analyzed the data with CGH-Plotter v2, and interpolated the values using window size 750 kBp. We identified several known copy number alterations within the data, and additionally many novel ones were detected [15,16].

With these data samples, we additionally tested the properties of interpolation by randomizing the genes to be removed from the 38 samples. Originally, there were CGH-values for 6487 genes in the samples. The number of removed values was set from 5% to 50% of all gene values in the sample. Thereafter from 340 to 3400 randomized values were removed from each sample. The data with missing values was smoothed with window sizes from 100 k to 1,000 k basepair units for finding out how the size of the window and the amount of the removed values effects on the filtering results. Thus, our test case data covered together 100 datasets, each with 38 samples.

**Figure 3:**
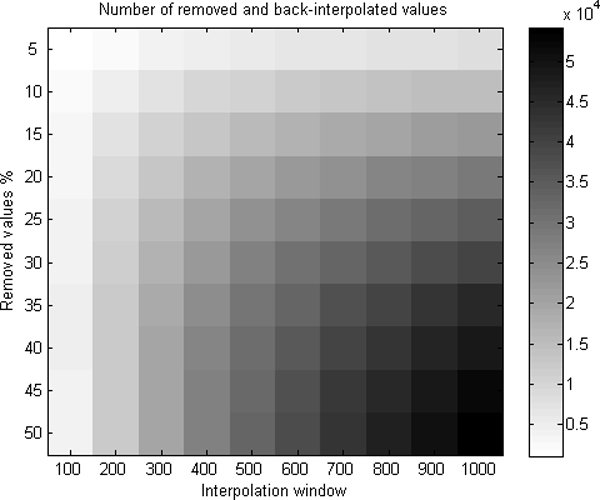
**The numbers of removed and back-interpolated values**. Depending on the percentage of removed values, the locations of them, and the window used in interpolation, the number of removed and back-interpolated values varied from 1,090 to 54,195, which is 0.4% to 22.0% of all values.

Further, we interpolated back the values with the same window size. The known gaps were input to the analysis as well. The interpolation of values for removed genes was done based on their location. The location of the randomly removed gene determined whether the gene value was interpolated or not. With interpolation, we could re-compute values for 0.4% to 22.0% of the genes when 5% to 50% were removed and the window size from 100 k to 1000 k basepair units was used in interpolation (Figure 3). The bigger was the size of the window, the more values got a value in interpolation.

**Figure 4:**
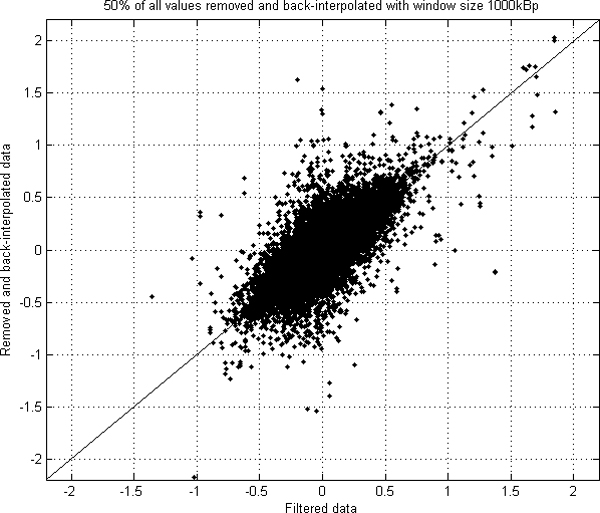
**The real filtered data plotted against removed and back-interpolated data**. The original data from 38 samples smoothed with genomic location based median filter with window size 1,000 kBp is in x-axis. 50% of the original values were randomly removed, and then back-interpolated with 1,000 kBp window. These interpolated values are in y-axis. The correlation between the original filtered and 50% removed and back-interpolated values with interpolation window size 1,000 kBp is 0.79.

**Figure 5:**
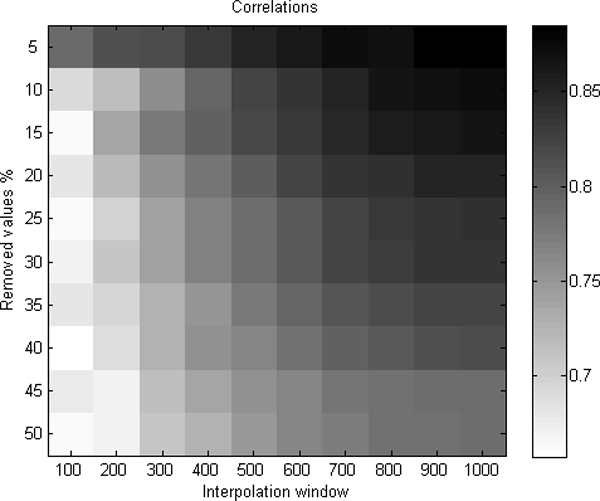
**The correlations between real filtered and removed and then back-interpolated values of 38 samples**. The larger was the window size and the smaller the number of removed values, the better was the correlation. Correlations varied from 0.66 to 0.89.

We computed the correlation of the values between the smoothed real data and the back-interpolated data (Figure 4). The correlation values between the data varied from 0.66 to 0.89 depending on the window size and percentage of removed values (Figure 5). However, the differences between correlations were not found to be significant, and the used value should be considered based on each data specifically depending on the resolutions of microarrays in use.

## Discussion

The analysis of both gene expression and gene copy number data is very important and interesting field in cancer research. The integrated copy number and expression analysis provides essential information about the behaviour of the genes in development of cancer. We developed a CGH-Plotter v2 for analyzing the copy number data. In addition, we created a fully compatible ECN-tool for illustrating the copy number and gene expression data. Both CGH-Plotter v2 and ECN-tool are easy-to-use, graphical interfaces and they require MATLAB 7 or higher to operate.

With CGH-Plotter v2 and ECN-tool it is possible to analyse and illustrate only those values that are present in the both microarrays. Additionally in CGH-Plotter v2, there is an option to infer the copy number values of the genes that are not measured with the microarray. The values are computed with interpolation done based on the genes locating next to the missing genes. Hence, the integrative illustration of the copy number and gene expression values can be performed with ECN-tool also from the copy number data including these interpolated values.

CGH-Plotter v2 and ECN-tool has been used for example in analysis of data from head and neck cancer [15] and oral tongue cell lines [16]. As a result of CGH data analysis, several known amplifications and deletions were identified with CGH-Plotter v2, and additionally many novel alterations were found. We performed a test study with 38 samples [15,16]. We tested the interpolated values with correlations computed between the filtered values of the real data, and the removed and back-interpolated data values by changing the filtering and interpolating window size and the percentage of removed values.

With CGH-Plotter v2 and ECN-tool it is easy to find both over-expressed and amplified, or under-expressed and deleted genes. Copy number variation is a crucial factor affecting gene expression. However, there are also other regulatory factors. Nevertheless, the list of identified associations between gene copy number and gene expressions is a good starting point for assessing the impact of copy number variation to gene expression, and the most interested associations may further be studied with wet-lab experiments.

## Conclusion

CGH-Plotter v2 and ECN-tool are easy-to-use graphical interfaces for analyzing and plotting copy number values together with gene expression values. Since the microarrays used for gene expression and gene copy number measurements are often different, including different sets of genes, the direct comparison between gene expression and gene copy number values is not possible. For these genes that have an expression value, but no copy number value measured, the copy numbers can be interpolated based on the neighbour genes. Since the copy number alterations usually span over larger regions, the interpolated copy numbers have a high correlations with the near ones, when the size of interpolation window is well selected. With ECN-tool the genes that are both amplified and over-expressed or deleted and under-expressed can easily be identified.

We evaluated the best window size to be used for interpolation of missing values. It was found that the best results are obtained when 15% or less of the data values are missing, and the used window size for smoothing and interpolating the data is larger than 600 k basepair units. Both versions of CGH-Plotter and ECN-tool can be downloaded at the website [17].

## Methods

### Dataset

In our case study we utilized a dataset with 38 samples [15,16]. The CGH experiments were performed with cDNA arrays on Human 1 cDNA microarray slides (Agilent Technologies, Palo Alto, CA, USA). The theoretical resolution of the CGH-arrays was 400 kBp. Also gene expression values were measured with microarrays from each sample. Expression analysis was performed on Agilent's Human 1A oligo array, which includes 13,643 unique genes. Thus, the theoretical resolution of the gene expression arrays was 225 kBp. Thus, the CGH-data of the study needed to be interpolated. After interpolation with window size 750 kBp, 11,761 genes had both copy number and gene expression value [15,16].

### Filtering

Let *I*_*l *_be a continuous interval in the genome, within there are no known gaps, *x*_*l *_be the value of gene in location *l*, and *l *∈ *I*_*l*_. Now the filtered value for gene *g*_*k *_in location *k *is

$$y_{k}=\text{median}\left(x_{l}|\max\left\{k-\frac{w}{2},\min\left(I_{l}\right)\right\}\leq l\leq \min\left\{k-\frac{w}{2},\max\left(I_{l}\right)\right\}\right),$$

where *w *is the size of the filtering window where the copy number values are allowed to have an effect on each other.

### Linear interpolation

The copy number value *y*_*g *_for gene *g *in location *bp*_*g *_can be computed by

$$y_{g}=y_{i}+\left(bp_{g}-bp_{i}\right)\frac{\left(y_{j}-y_{i}\right)}{\left(bp_{j}-bp_{i}\right)},$$

where *y*_*j *_is the filtered copy number of gene in location *bp*_*j *_and *y*_*i *_is the filtered copy number of gene in location *bp*_*i*_. Further,

*bp*_*i *_<*bp*_*g *_<*bp*_*j*_,

and in order the interpolation to take place, there must be no known gaps between genes *i *and *j*, and the interpolation window *w *is needs to be set to

*w *≥ 2·max {*bp*_*g *_- *bp*_*i*_, *bp*_*j *_-*bp*_*g*_}.

This requirement ascertains that the genes next to the missing one align within the filtering window and a continuous area, and genes separated by known gaps in genome, will not have an effect on each other (Figure 2). Therefore, as well as the gene location based median or mean filtering process, interpolation process will not interpolate over known gaps.

## Competing interests

The authors declare that they have no competing interests.

## Authors' contributions

RA and MS wrote the codes and web pages of the CGH-Plotter v2, and ECN tool. SH wrote the original version for functions to plot CGH-values annotated based on gene expression. AKJ, RA and MS selected the methods needed in CGH-Plotter v2 and ECN tool. JA supervised the project and RA wrote the manuscript. All authors read and approved the final manuscript.
